# Leg Length Discrepancy and Nonspecific Low Back Pain: 3-D Stereophotogrammetric Quantitative Posture Evaluation Confirms Positive Effects of Customized Heel-Lift Orthotics

**DOI:** 10.3389/fbioe.2021.743132

**Published:** 2022-02-10

**Authors:** Moreno D’Amico, Edyta Kinel, Piero Roncoletta

**Affiliations:** ^1^ SMART (Skeleton Movement Analysis and Advanced Rehabilitation Technologies) LAB, Bioengineering and Biomedicine Company Srl, Chieti (CH), Italy; ^2^ Department of Neuroscience, Imaging and Clinical Sciences University G. D’Annunzio, Chieti, Italy; ^3^ Chair of Rehabilitation and Physiotherapy, Department of Rehabilitation, University of Medical Sciences, Poznań, Poland

**Keywords:** leg length discrepancy, nonspecific low back pain, stereophotogrammetry, posture, spine, customized orthotics

## Abstract

**Background:** The literature reports evidence of leg length discrepancy (LLD) associated with musculoskeletal disorders, alterations in spinopelvic alignment, and body posture, leading to low back pain and lumbar scoliosis. The most common conservative treatment for LLD is the use of internal or external shoe lifts although no treatment guidelines have been established.

**Aim:** The study aimed to contribute to low back pain–LLD relationship comprehension, highlighting the benefits of LLD correction in the nonspecific low back pain (NSLBP) population.

**Methods:** A cross-sectional observational study recruited a cohort of 80 NSLBP patients (48 females, 32 males) with LLD, age (*μ* = 35 ± 17.2). Entire body posture, including 3-D spine shape reconstruction, was measured using a nonionizing 3-D optoelectronic stereophotogrammetric approach. After the first 3-D posture evaluation, patients were provided with customized orthotics, including 100% LLD heel lift correction. No other therapeutic interventions were considered. Pain level was assessed using the numerical pain rating scale (NPRS). The gender, age-related, and time-dependent effects of LLD equalization treatment in NSLBP patients was investigated during 2 years of follow-up. The statistical analysis was performed at the global level using multivariate methods by Hotelling *T* ^2^ tests and intrasubject-level using *t*-test.

**Results and Discussion:** An initial average NPRS = 7.8 was determined. In the medium-term follow-up group (4 months), the NPRS dramatically decreased (NPRS = 1.1). The pain disappeared in the long-term (2 years) follow-up group (NPRS = 0). The study results highlight that LLD equalization treatment led to clear statistically significant improvements in all the postural parameters of the frontal plane, including the underfoot load asymmetry. No worsening has been detected. An adaptation period long enough is needed to obtain progressive pain relief improvements and structural posture changes. Younger NSLBP patients showed slightly better improvements than older ones. Minimal differences between healthy young adults’ and NSLBP patients’ postures were found either in natural erect standing posture or when LLD equalization is applied.

**Conclusion:** Heel-lift customized orthotics with 100% LLD correction are an effective short- and long-term treatment in patients with nonspecific LBP, inducing pain symptom recession and stimulating the improvement of postural parameters without contraindications.

## 1 Introduction

Anisomelia, i.e., limb length discrepancy, is defined as a condition in which paired limbs are noticeably unequal. When the discrepancy is in the lower extremities, it is known as leg length discrepancy (LLD). Recent literature reports increasing evidence of the association of LLD with a variety of musculoskeletal disorders, such as hip or knee osteoarthritis and chronic low back pain (LBP) that impose a high personal and social burden (Defrin et al., 2005; Tallroth et al., 2005; Harvey et al., 2010; Kendall et al., 2014; Murray and Azari, 2015; Tallroth et al., 2017; Gordon and Davis, 2019). Perhaps the most controversial musculoskeletal disorder associated with LLD is LBP. Some authors find a definite association between LLD and LBP (Friberg, 1983; Defrin et al., 2005; Golightly et al., 2007; Kendall et al., 2014; Cambron et al., 2017; Rannisto et al., 2019; Menez et al., 2021; Menez et al., 2020), whereas others find none (Campbell et al., 2018). LLD can be either anatomical, i.e., caused by deformities originating from actual differences in the bony structures of the lower limb, or functional, i.e., possibly derived by mechanical changes affecting the lower limbs (Blake and Ferguson, 1992; Brady et al., 2003; Knutson, 2005a; Knutson, 2005b). However, there is still a lack of agreement on diagnosis (Brady et al., 2003), classification (Gurney, 2002), and treatment (Campbell et al., 2018) among both researchers and clinicians. The existence of limb length inequality is not in doubt. However, its prevalence in the population constitutes an open debate because there is no agreement on what constitutes a clinically significant LLD (Brady et al., 2003). In most cases, the coexistence of anatomical and functional contributions to LLD adds difficulties to the LLD evaluation. An extensive review by Sabharwal and Kumar (2008) shows no agreement on how the LLD has to be measured with either clinical or instrumental methods. Clinical methods, such as the use of a tape measure and standing blocks, (being the latter to be preferred in that they were found to be more reliable and complete giving the possibility to consider also the LLD functional component) are noted as useful screening tools, but not as accurate as imaging modalities. However, even on imaging tools to be used, there is a debate, and they present pros and cons, especially thinking about the risk connected to the use of X-rays for some of them.

Since the early ’80s, the literature describes it as a relatively common phenomenon found in as many as 40% (Subotnick, 1981) to 70% (Woerman and Binder-Macleod, 1984) up to 90% of the adult population (Blake and Ferguson, 1992), whereas the incidence of LLD greater than 20 mm was found to affect at least one in every 1000 people (Guichet et al., 1991). Later on, LLD has been recognized to affect up to 90% of the population with an average value of 5.2 ± 4.1 mm as measured by highly precise radiographic millimetric methods (Knutson, 2005a). More recently, D’Amico et al. (2017b) reported the normative 3-D posture and spine shape data in a healthy young adult population. Using an advanced optoelectronic stereophotogrammetric approach to measure the entire 3-D skeleton posture, including 3-D spine morphology during natural, unconstrained standing erect posture, they found, in a cohort of 124 asymptomatic volunteers, that each subject presented some degree of LLD. Despite male and female groups presenting statistically different heights, the amount of LLD was gender-independent with a mean value *µ* = 9.37 ± 3.31 mm and in a range of 6–23 mm. LLD was associated with the following factors: unbalanced underfoot loads and/or posture, some amount of spinal deformity and pelvis obliquity in the frontal plane, and pelvis torsion. Some authors classify discrepancies ≤2.0 cm as mild (Ruzbarsky et al., 2017), whereas others consider discrepancies of up to 3.0 cm as mild (Gurney, 2002; Brady et al., 2003; Campbell et al., 2018). A subdivision into mild (up to 30 mm), moderate (30–60 mm), or severe (>60 mm) is also reported (Reid and Smith, 1984; Brady et al., 2003; Knutson, 2005a). These classifications are intended to guide practitioners in treating LLD (Vogt et al., 2020), but there is much disagreement in the literature about the magnitude from which LLD requires treatment. Some authors may consider that LLDs less than 2 cm are usually well-tolerated and often go unnoticed (Ruzbarsky et al., 2017). Conversely, it is suggested that orthotic insoles, shoe lifts, or other clinical interventions to equalize leg length should be considered for LLD ≥1.0 cm (White et al., 2004) or even between 0.5 and 1.0 cm (Friberg, 1982; Friberg, 1983; Khamis and Carmeli, 2018; Gordon and Davis, 2019; Menez et al., 2021; Menez et al., 2020).

LLD is associated with alterations in spinopelvic alignment (Knutson, 2005a; Cooperstein, 2010), body posture (Raczkowski et al., 2010; Murray and Azari, 2015; D’Amico et al., 2017b), and balance (D’Amico et al., 2017b; Eliks et al., 2017), thus determining an asymmetrical distribution and magnitude of mechanical stresses and strains within the body (McCaw and Bates, 1991). Particular attention is devoted to LLD effects on pelvic posture and motion. Several authors notice pelvic tilt in the frontal plane due to uneven leveling of lower limbs associated with a forward innominate bone rotation on the short limb and backward rotation on the long limb (McCaw and Bates, 1991; Cooperstein, 2010). Such a complex destabilizing in the pelvis (i.e., pelvic obliquity associated with pelvis torsion) is found to relate to the lower limb loading pattern for healthy, young, asymptomatic individuals. Indeed, D’Amico et al. (2017b) shows a higher probability that longer-leg loaders demonstrate more posterior rotation on the ipsilateral side than shorter-leg loaders who would more likely show anterior rotation on this side. Numerous observational studies reveal correlations between LLD, asymmetrical distribution, and magnitude of mechanical stresses and strains in spine joints, degenerative changes in the lumbar spine, alterations in spinal biomechanics, and LBP. However, they fail to show causation, resulting in limited evidence to guide treatment (Sheha et al., 2018). A certain magnitude of LLD likely plays a role in LBP although it is unclear at this time what degree of LLD is required to cause symptoms. Indeed, given changes in multiple parameters that tend to occur with LLD (e.g., sacral or pelvic tilt and lumbar scoliosis, compensations in the lower limbs), it is likely that confounders are at play. Therefore, the exact relationship linking the LLD, the lumbar spine, and the true drivers of LBP in these patients has yet to be fully elucidated (Sheha et al., 2018).

Treatment of LLD is an open debate in the literature. It spans from no intervention to conservative approaches to various surgical techniques (Gurney, 2002; Brady et al., 2003). Conclusions in a recent review (Vogt et al., 2020) recommend the following: “It must be discussed with each patient individually whether the treatment should be conservative or surgical. The extent of the discrepancy is not the sole determining factor for the mode of treatment. The decision to treat is always elective.”

The most common conservative treatment for mild LLD is the use of internal or external shoe lifts (Brady et al., 2003). Even on such a simple intervention, there are a plethora of opinions regarding shoe lift efficacy, the best thickness to be used, and even the strategies for their application (Brady et al., 2003; Golightly et al., 2007; D’Amico et al., 2012; Chuter et al., 2014). Many are the studies reporting benefits of shoe insert use inducing symmetrical limb loading, pain, and functional disability reduction and lumbar scoliotic curve reduction (Friberg, 1983; Defrin et al., 2005; Golightly et al., 2007; D’Amico et al., 2012; Chuter et al., 2014). Conversely, others find limited data to support their use (Brady et al., 2003; Chuter et al., 2014).

In this context of uncertainty, the present research aims to study the LBP–LLD relationship. Our primary hypothesis is that the uncertainty debated in the literature is mainly due to the lack of detailed, rigorous, biomechanical-functional information on 3-D natural erect standing posture characteristics and how the presence of an LLD influences them.

To tackle the several confounders at play, we use the nonionizing optoelectronic stereophotogrammetric approach associated with baropodometry presented in D’Amico et al. (2017a, 2017b). Such a measurement technique shows several advantages, allowing, in a very short time, the evaluation of the 3-D entire skeleton posture, including the 3-D spine shape, analyzed with many clinically useful 3-D and 2-D anatomical, biomechanical, and clinical parameters. In this way, it is easy and fast to quantify the eventual presence of an LLD and the whole skeleton functional postural adjustments when a heel-lift LLD correction is applied. At the same time, the optimal LLD correction can be immediately identified by evaluating the effects of different thickness heel lifts on the patient’s posture by statistically comparing the LLD equalized erect posture with the actual neutral unconstrained upright standing. We collected data on a cohort of nonspecific low back pain (NSLBP) patients presenting an LLD to investigate the immediate, medium, and long-term answers of LLD equalization considering the effects on different age groups. Finally, to identify whether there are specific 3-D postural characteristics for the NSLBP population and specific behavior of such population in response to LLD equalization, we compared the results obtained in the present study with those obtained for a population of healthy young adults determined in the study of D’Amico et al. (2017b).

## 2 Materials and Methods

### 2.1 Study Design

The present study is a prospective, cross-sectional observational research (according to the STROBE guidelines (von Elm et al., 2007) and the Helsinki Declaration), evaluating the LLD equalization effect through custom-made foot orthotics in nonspecific chronic and subacute LBP patients using 3-D stereophotogrammetric quantitative posture analysis. The ethics committee of the University of Medical Sciences in Poznan, Poland, approved this study (resolution number: 376/17). Written informed consent was obtained from the individuals for the publication of any potentially identifiable images or data included in this article. Data collection took place between May 2017 and December 2019.

### 2.2 Participants

Before the measurement session, participants were given a thorough clinical postural examination by an experienced physiotherapist, during which pain intensity was rated via the numerical pain rating scale (NPRS) (Jensen and Karoly, 2001).

The inclusion/exclusion criteria were as follows: diagnosis of NSLBP both subacute (≥6 weeks) and chronic (≥12 weeks) nonspecific lumbar pain history, males and females older than 18 years (Caucasian), no neurologic problems, no history of musculoskeletal system injury or surgery, LLD presence measurable through the used stereophotogrammetric approach.

A cohort of 80 NSLBP patients (48 females, 32 males), 18–72 years (*μ* = 35 ± 17.2) were recruited in a cross-sectional observational study at the Clinic of Rehabilitation, University of Medical Sciences, Poznan-Poland. Table 1 summarizes the characteristics of the NSLBP patients compared by gender to healthy young adults.

**TABLE 1:** NSLBP Patients Characteristics Compared to Healthy Young Adults Characteristics. The last column reports the comparison between males and female characteristics in the NSLBP patients’ cohort (total = 80 NSLBP patients).

	NSLBP mean (SD)	HYAP mean (SD)	*t*-Test NSLBP vs HYAP	*t*-Test NSLBP females vs males
Age (yr)^a^	35.0 ± 17.2	24.25 ± 3.6	*p* < 0.001	—
Age males (yr)	33.9 ± 18.2	24.9 ± 3.9	*p* < 0.001	ns
Age females (yr)	36.6 ± 16.5	23.5 ± 3.2	*p* < 0.001	
Weight males (kg)	72.6 ± 11.9	73.9 ± 9.3	ns	*p* = 7.0e-21
Weight females (kg)	61.1 ± 9.7	57.7 ± 9.1	ns	
Height males (cm)	175.5 ± 7.1	178.3 ± 6.5	ns	*p* = 6.7e-10
Height females (cm)	163.4 ± 6.5	164.3 ± 5.3	ns	
BMI males (kg/m^2^)	23.5 ± 3.3	23.2 ± 2.1	ns	ns
BMI females (kg/m^2^)	22.9 ± 3.6	21.3 ± 2.6	*p* = 0.013	

a Total NSLBP, patients Age Range = 18–72 years; Total Healthy Young Adults Age Range = 19–35 years.

ns*= not significant*.

### 2.3 Data Measurement

Patients’ entire body posture, including 3-D spine shape reconstruction, has been measured using a nonionizing 3-D optoelectronic stereophotogrammetric approach. Eleven quantitative biomechanical parameters describing the nature of body posture have been computed. Our experimental recordings were based on a six-TV-camera Global Opto-electronic Approach for Locomotion and Spine^1^ (GOALS) stereophotogrammetric optoelectronic system derived from Optitrack System^2^ [resolution 1.3 Mpix, 120 fps, error range 0.3 mm, calibrated volume 3 × 3 × 2 meters (D’Amico et al., 2017b; D’Amico et al., 2018a; Kinel et al., 2018)]. One synchronous baropodometric platform (Zebris FDM-SX)^3^ (active area dimensions: 400 mm × 330 mm, total 1920 square capacitive sensors; 1.4 sensors/cm^2^) was used to measure bilateral foot pressure maps and underfoot vertical forces exerted on each foot in the standing position. The platform is rectangular, and the sensors are arranged in rows and columns parallel to the shorter and longer edge, respectively. The manufacturer grants the calibrated pressure measuring range (1–120 N/cm^2^) with ±5% of the maximum range accuracy. An essential step of the calibration procedure is to establish the relative position of the baropodometric platform within the calibrated volume. This position is essential for all the following calculations related to underfoot pressure maps and associated vertical forces. Further details about the calibration procedure can be found in D’Amico et al. (2021a).

**FIGURE 1:**
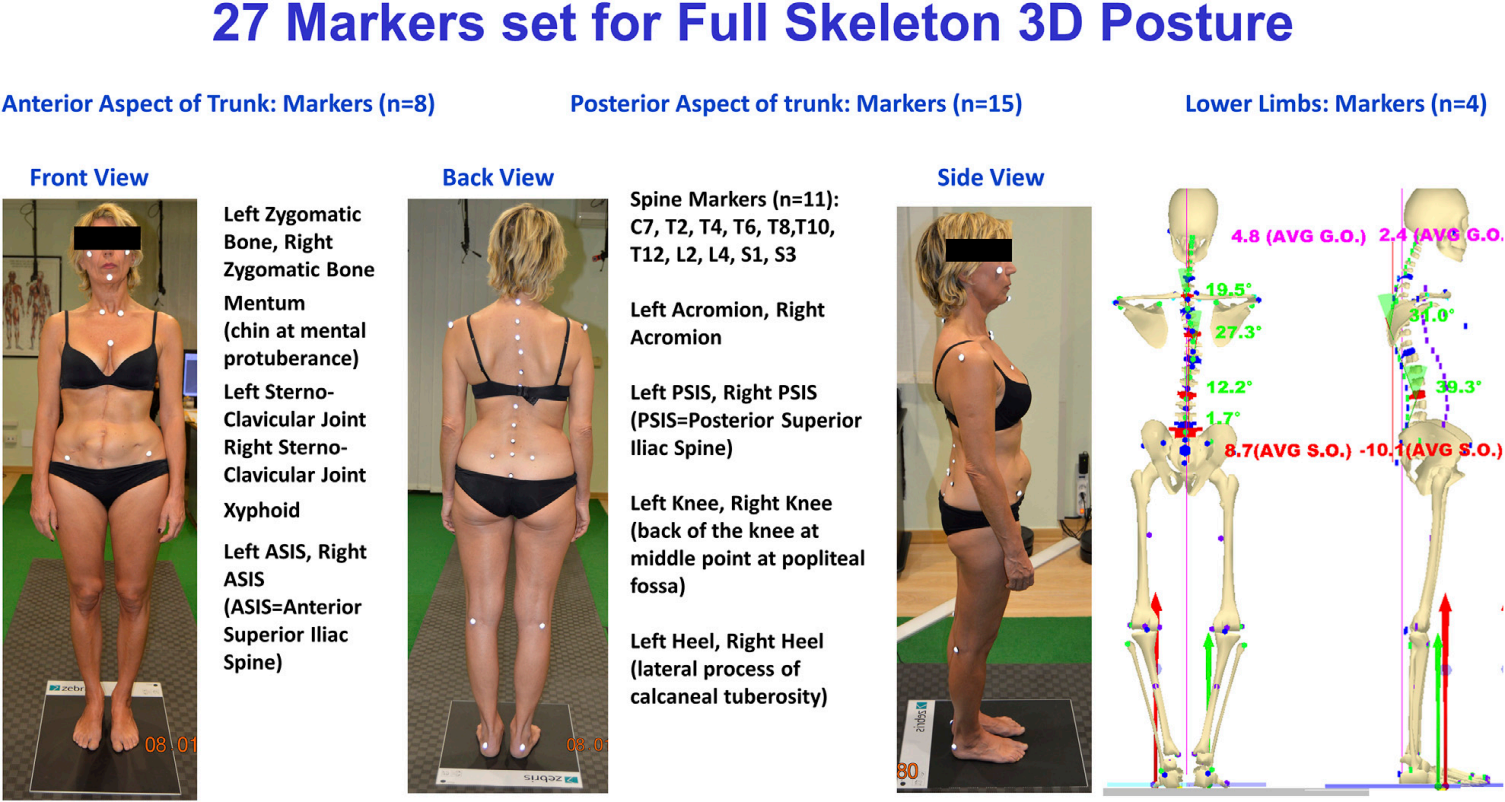
The experimental setup used for 3-D posture analysis: GOALS system and baropodometric platform configuration.

The 27 body landmarks protocol, labeled by passive retroreflective markers (D’Amico et al., 2018b), was used to measure the subjects’ 3-D whole skeleton posture (Figure 1). The model’s accuracy and precision are founded on in-house original signal processing and optimization procedures (D’Amico et al., 1995a; D’Amico et al., 2007; D’Amico et al., 2021a) and anatomical studies listed in the literature (cadaver dissections, *in vivo* X-ray, and parametric regression equations from gamma-ray measurements) (Liu and Wickstrom, 1973; Zatsiorsky et al., 1990; Seidel et al., 1995; de Leva, 1996). The model was formulated in a parametric form to scale any subject’s characteristics by fitting each given skeletal segment to the 3-D measured positions of its corresponding body landmarks (D’Amico et al., 1995a). The model was tested extensively in the clinical environment to analyze human posture (D’Amico et al., 2017a; D’Amico et al., 2017b; D’Amico et al., 2018a; Kinel et al., 2018; D’Amico et al., 2021a; Kinel et al., 2021).

The software package called ASAP 3D Skeleton Model,^1^ implementing a full 3-D parametric biomechanical human skeleton model (3-D spine included), was used to process data.

### 2.4 Acquisition Protocol

The standard trial session aimed to define the participant’s erect posture. In order to reduce potential postural effects resulting from circadian rhythms, all measurements were taken between 12 noon and 7 pm. The subjects were asked to avoid training/therapeutic exercise and/or demanding physical activity before the postural assessment. The assessment/measurement session aimed to fully capture and record the subject’s neutral standing posture with the upper arms relaxed along the side of the body and eyes looking directly ahead in the horizontal plane. Such posture has been defined as indifferent orthostasis (IO). For all subjects, marker positioning was performed by a single operator with more than 20 years’ experience. Each marker-positioning session was of approximately 10 min duration, after which the subject was asked to sit for a few minutes. Afterward, the subject was asked to keep an IO standing with both feet on the baropodometric platform (D’Amico et al., 2017b; Kinel et al., 2018).

Different positions of the feet can influence standing postures. Thus, the subject was asked to align heels on a line parallel to the frontal plane (i.e., on a line parallel to the *X*-axis of the laboratory reference system) and keep feet apart at about pelvis width (i.e., with feet under the hip joints’ projection) without restricting feet direction to avoid feet position influence. Real-time baropodometric measurement availability allows controlling feet alignment straightforwardly by checking that the heels’ most prominent tip lay on the same row in the foot pressure maps. The subject was positioned in front of a blank wall to avoid visual feedback or reference during measurements.

Given the inclusion criteria, every subject presented with some degree of LLD. In other words, a difference in right vs. left hip center heights as assessed by anterior and posterior iliac spine (ASIS and PSIS) landmark positions (D’Amico, 2002; D’Amico et al., 2012; D’Amico et al., 2017b). Therefore, an additional measurement was then conducted following the same protocol as for IO but by placing a suitable wedge under the subject’s shorter leg as a corrective to equalize any found LLD. Such a measurement was called wedge-corrected orthostasis (WCO). In order to take into account both “anatomical” and “functional” LLD (Knutson, 2005a), it was decided that the optimal size for the corrective wedge was the degree of thickness that provided the best outcome for the individual subject concerning frontal-plane postural parameters (see below). If an overall improvement of the frontal-plane postural parameters was not obtained, the optimal corrective-wedge thickness was chosen as the one providing the best equalization regarding the PSIS landmarks (D’Amico et al., 2017b). In general, no more than three subsequent WCO measurements were necessary to establish the wedge optimal thickness. Such optimal thickness is considered the LLD value in the subsequent statistical computations.

Between each series of measurements (IO and the subsequent WCO trials), the patient was free to sit and relax for a few minutes. The entire acquisition session for each subject was generally completed within 30 min from the time of the subject’s arrival at the posture analysis laboratory, through the complete assessment process, to the final biomechanical report containing a complete set of IO plus WCO measures.

At least five subsequent 2-s lasting acquisitions at a sampling rate of 120 Hz were recorded for each IO and WCO attitude. A total of 1200 3-D measurements for each static posture was averaged. Before averaging, an amount of preprocessing is needed on the acquired 3-D raw data to define the subject’s local coordinate system and its orientation relative to the global coordinate system (D’Amico et al., 2017a; D’Amico et al., 2017b; D’Amico et al., 2018a; Kinel et al., 2018). We used the general definitions provided by the Scoliosis Research Society (Stokes, 1994). However, in distinction to such recommendations, PSIS rather than ASIS landmarks are considered in defining the subject’s local coordinate system to reduce propagation errors and/or other interference deriving from pelvis torsion in the subsequent calculation of spinal parameters. Once having determined this individual system, a rotation is performed within each frame to align the subject’s coordinates with the global reference coordinates. When the alignment is complete, it is possible to average all acquired frames properly. The complete analytical and mathematical descriptions of the entire procedure to reconstruct the whole 3-D skeleton are beyond this paper’s aim, wherein only the main features of 3-D spine reconstruction are described. Full details can be found in D’Amico et al. (1995a,2017a, 2017b, 2018a, 2021a) and Kinel et al. (2018, 2021).

Based on the 11 3-D spinous process measurements, data are interpolated using cubic splines (de Boor, 2001) to assess the position of each unlabeled spinous process and intervertebral disc. After interpolation, the space-curve modeling of the spine is analytically represented using three parametric functions *x*(*t*), *y*(*t*), *z*(*t*) (the parameter being *t* > 0). A smoothing and differentiation procedure specially developed for interpolated data with cubic splines is applied to these functions (D’Amico et al., 1995a; D’Amico et al., 2017b; D’Amico et al., 2021a). Once the three parametric functions *x*(*t*), *y*(*t*), *z*(*t*) and their derivatives are assessed, the 3-D position of each vertebra from C7 down to S3 is derived. The maxima and minima of the assessed first derivative allow selecting, under analytical constraint, all the inflection points defining the limit vertebrae. After determining the limit vertebrae, the Cobb and kypho-lordotic angle computation is straightforward, computing the angle between the tangents in such points (D’Amico et al., 1995a; D’Amico et al., 2017a; D’Amico et al., 2017b; Kinel et al., 2018). Worth noting, as it happens for the curve identified in the frontal plane, also the kyphosis and lordosis in the sagittal plane are appropriately identified according to the actual spine curvature spatial changes at the limit-vertebrae; i.e., they are no longer restricted to specific thoracic or lumbar anatomical regions (D’Amico et al., 2017b). The accuracy and precision of such signal processing procedures are demonstrated through numerical simulation (D’Amico et al., 2021a), showing excellent performance. In particular, using 3-D analytical helixes, it is demonstrated that, when white noise stereophotogrammetric error (*σ* = 0.3 mm) was superimposed, the mean angle error computation was only a fraction of degree (0.65°) on a deformity angle up to 66°. Interestingly, the algorithm showed a substantial insensibility to the marker misplacement error (added to the noisy helix *σ* = 5 mm) along the longitudinal direction. In this case, the resulting mean estimation error presented the same magnitude (0.68°) as the previous test. Finally, when the added misplacement error was considered along random 3-D positions, the computed mean error resulted in 2.52° (D’Amico et al., 2021a).

**FIGURE 2:**
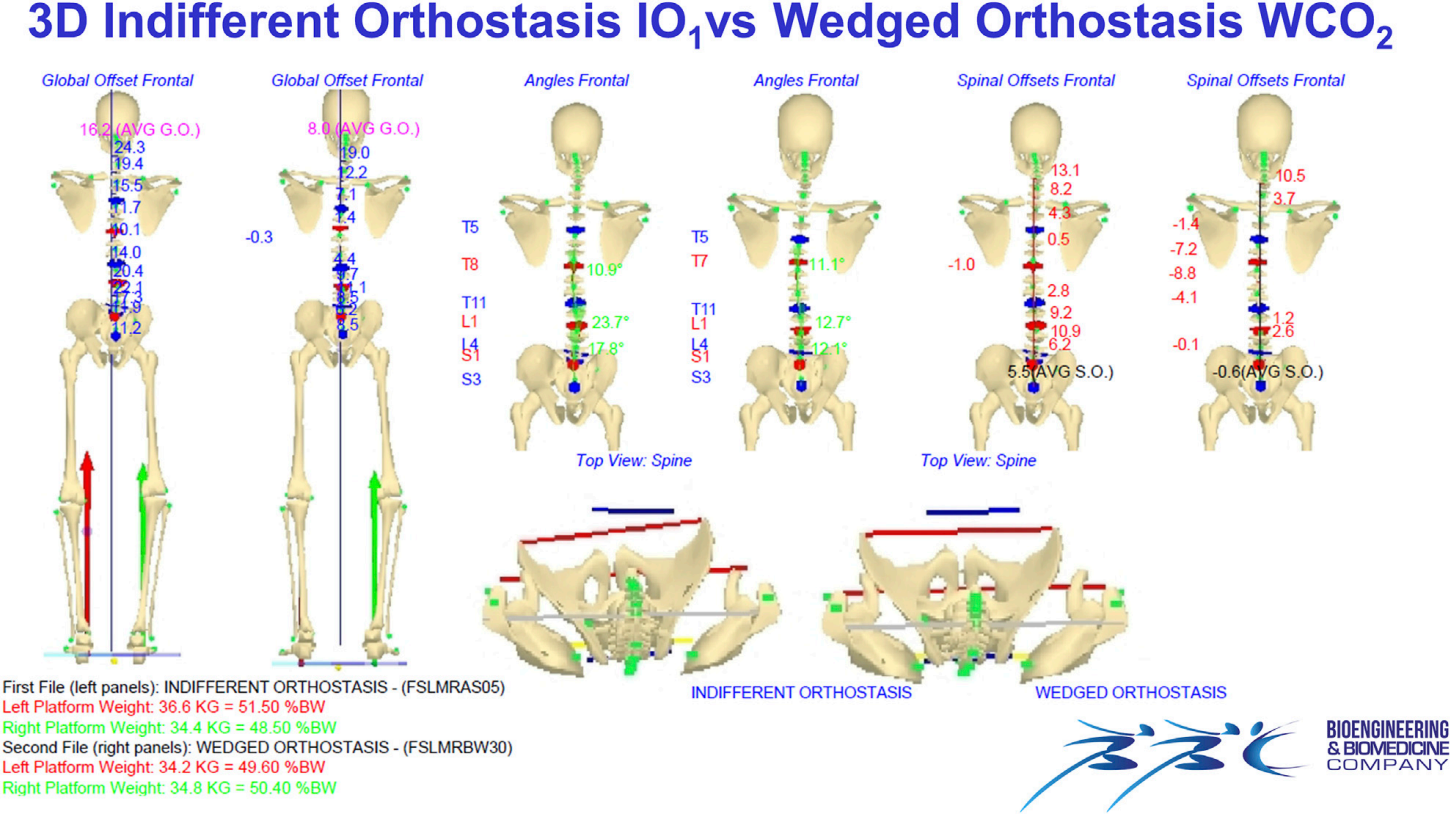
Example of data elaboration outcome and the related graphical report of the IO_1_ vs. WCO_2_ measurement comparison in the frontal (Panel 2) and sagittal (Figure 3) planes.

**FIGURE 3:**
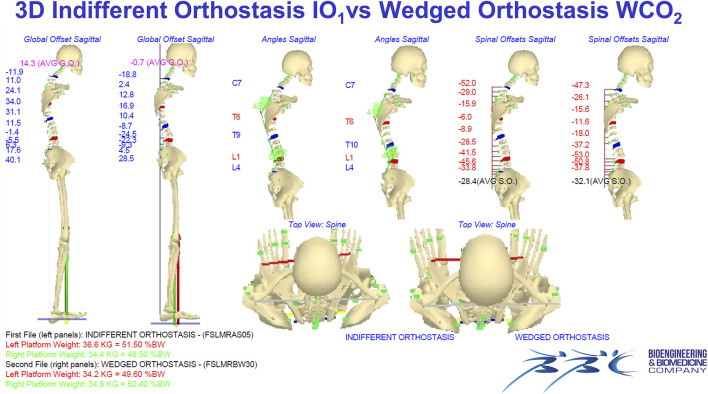
Example of data elaboration outcome and the related graphical report of the IO_1_ vs. WCO_2_ measurement comparison in the frontal (Figure 2) and sagittal (Panel 3) planes. IO_1_ (IO at first evaluation); WCO_2_ (WCO at control session).

Figures 2, 3 Example of data elaboration outcome and the related graphical report of the IO_1_ vs. WCO_2_ measurement comparison in the frontal (Figure 2) and sagittal (Figure 3) planes.

A video showing the acquisition/elaboration processes can be found in the supplementary material (Supplementary Material Video S1).

A set of 11 significant parameters detailing the 3-D body posture structure is computed from the 3-D biomechanical human skeleton model reconstruction (D’Amico et al., 2017b; D’Amico et al., 2018a). Such variables were subdivided into three groups as reported in Table 2.

**TABLE 2:** List of considered parameters (definitions and corresponding acronyms) for IO vs. WCO comparison and Summarizing Indexes.

Global summarizing index	Parameters			Specific summarizing indexes
GPI global postural index	**Acronyms**	**Descriptions**	**Definitions**	
	|ASO FR| (mm)	|Average frontal spinal offsets|	The ASO is the mean of the horizontal distances in the frontal plane of each labelled spine landmark respect to the vertical axis passing by S3; Absolute value of the average to disregard the side	FPI (Frontal Postural Index)
	|AGO FR| (mm)	|Average frontal global offsets|	The AGO is the mean of the horizontal distances in the frontal plane of each labeled spine landmark respect to the vertical axis passing through the middle point between heels; Absolute value of the average to disregard the side	
	|∆ASIS| (mm)	|∆Anterior superior iliac spine|	Absolute ASIS height difference in frontal plane	
	|∆PSIS| (mm)	|∆Posterior superior iliac spine|	Absolute PSIS height difference in frontal plane	
	CA1; CA2 (degrees)	1° Cobb angle; 2° Cobb angles	Cobb angles of the two main “spinal deformities” found in the frontal plane		
	|PT|mm	|Pelvis torsion| = |(∆ASIS–∆PSIS)|	Rotation of the Right respect to the Left Innominate bone. Rotations are intended around a horizontal axis running through the symphysis pubis. Absolute value to disregard the side	SPI (Sagittal Postural Index)
	SA (degrees)	Sacral angle	The inclination of S1-S3 line respect to the vertical line	
	TKA (degrees)	“Thoracic” kyphosis angles	Kyphosis and Lordosis are correctly identified following spine curvature spatial changes at inflexion points, and so limit vertebrae are not strictly bounded to the specific anatomical region	
	LLA (degrees)	“Lumbar” lordosis angles	The inclination of S1-S3 line respect to the vertical line	
	|∆UL| (%BW)	|∆Underfoot Load|	Left vs. R=right sides body weight (BW) Percentage Difference. Absolute value to disregard the side	

We decided to consider the Cobb angle value of the two major curves (CA1, CA2, Table 2) for statistical analysis regarding the spinal deformities in the frontal plane.

### 2.5 Baropodometric Measurements and LLD Equalization by Customized Foot Orthotics

After the first evaluation session, each patient was provided with custom-made foot orthotics to be donned in their usual shoes. As described, during the stereophotogrammetric measurement of static erect postures, simultaneous baropodometric measurements were collected. After the wedge optimal thickness was determined, further baropodometric dynamic measurements were collected during gait trials to complete the information about dynamic foot-floor interaction for each patient’s foot. Indeed, in addition to LLD, each subject presented an individual foot-floor interaction given his/her foot shape and functional behavior (flat foot, arched foot, etc.). Worth underlining, individual behavior was analyzed per foot given the loading asymmetry characteristics linked to LLD (Menez et al., 2021; Menez et al., 2020). A 3-D foot scan complemented such information to proceed to manufacturing customized foot orthotics using a CAD-CAM milling technique as described in detail in D’Amico et al. (2021b). The materials used in the customized orthotics were ethinyl vinyl acetate hand-finished by applying a 1.5 mm PPT top cover. Thus, the customized foot orthotics were made according to the therapeutic needs of the subjects and incorporated a heel lift on the short leg side. The heel lifts were corrective of the 100% LLD (D’Amico et al., 2012) and were shaped from the calcaneus to the Lisfranc joint. No other therapeutic interventions were considered.

### 2.6 Group Statistical Analysis

The group statistical analysis was performed using a multivariate method provided the checked correlation (through the computation of correlation matrices) among the considered 11 quantitative postural parameters.

According to the specific test, all the following comparisons have been assessed using either the independent samples or the paired samples versions of Hotelling’s *T* ^2^ test. In the results section, the kind of test used is reported in the table caption.

The simultaneous 95% confidence intervals (derived from Hotelling’s *T* ^2^ tests) were undertaken to determine the statistical significance of the difference of means for each of the 11 quantitative parameters (Rencher, 2003). Such a method is preferable compared with setting a battery of separate *t*-tests for each variable with Bonferroni correction on the type I error (α’ = α/k) because the latter approach does not take into account the correlation between the variables, and therefore, it results in an overcorrection of the significance value α (Rencher, 2003).

#### 2.6.1 NSLBP Patients’ Postural Characteristics and Reactions to LLD Equalization, Evaluated by Gender

The following group comparisons have been performed: 1) two female vs. male comparisons, one in IO_1_ at first evaluation and the other in WCO_2_ at control sessions; 2) the IO_1_ vs WCO_1_ at first evaluation and IO_2_ vs. WCO_2_ at control sessions evaluated by gender; such comparisons provide information about the immediate-term postural reaction to LLD equalization and how the adaptation period influences such immediate-term answer; 3) the IO_1_ at first evaluation vs. IO_2_ at the control sessions evaluated by gender; 4) the IO_1_ at first evaluation vs. WCO_2_ at the control sessions evaluated by gender. The last two comparisons allow assessing structural postural changes induced by custom foot orthotics.

Moreover, to study time-dependent (medium and long-term) effects of LLD equalization induced by customized foot orthotics, the sample has been subdivided into two groups of equal size (24 females and 16 males). The IO_2_ and WCO_2_ were evaluated after 4 months in the medium-term group. The follow-up lasted up to 2 years for the long-term group when the final IO_2_ and WCO_2_ evaluation was performed. The NPRS (Jensen and Karoly, 2001) was readministered at the medium and long-term evaluations.

Finally, to highlight the age-dependent effects of LLD equalization induced by customized foot orthotics, the sample was subdivided into two groups: younger adults 18–40 years (51 total, 23 males 28 females) and older adults >40 years (29 total, 9 males 20 females).

Only the IO_1_ vs. WCO_2_ comparison has been considered for the time and age-dependent effects. The males and females have been pooled together to increase the test power and effect size.

#### 2.6.2 Differences Between NSLBP Patients and Healthy Young Adults in Postural Characteristics and Reactions to LLD Equalization, Evaluated by Gender

To study any differences between NSLBP patients compared with healthy young adults (measured in a previous study (D’Amico et al., 2017b)) in both basic postural characteristics and reactions when an LLD equalization is applied, the following series of comparisons by gender were performed: IO_1H_ vs. WCO_1H_, IO_1NSLBP_ vs. IO_1H_, WCO_1NSLBP_ vs. WCO_1H_, and finally WCO_2NSLBP_ vs. WCO_1H_. The subscript NSLBP and H indicate NSLBP and healthy young adults groups, whereas the subscripts 1 and 2 indicate, as above, the first or the control session.

Worth noting, control session was not considered for the healthy subjects in the study of (D’Amico et al., 2017b), in which only the immediate-term of LLD equalization by the application of a simple underfoot wedge of optimal thickness (see above) has been scrutinized. For consistency with the statistical approach followed in this study, the immediate-term answer of LLD equalization of the healthy young adults, i.e., the IO_1H_ vs. WCO_1H_ comparison, is performed applying the multivariate approach (*T* ^2^ Hotelling test for paired samples version) on the data published in (D’Amico et al., 2017b). Because the sacral angle (SA) parameter was not considered in the study of healthy young adults, all the comparisons involving the healthy subject group are performed considering the remaining 10 parameters.

#### 2.6.3 Correlation Between Heel Lift Thickness and Changes in the Main Cobb Angle (CA1)

The correlation between the changes of the main CA1 passing from IO_1_ to WCO_2_ and the heel lift correction determined at first evaluation has been assessed. As the data was not normally distributed, the Kendall’s Tau correlation was used.

### 2.7 Intrasubject Statistical Analysis

At the intrasubject level, we investigated how LLD equalization changed the subject’s posture as assessed in two different temporal circumstances: 1) immediate effect, i.e., when the measurement is made by comparing the within-session IO vs. WCO both at the first evaluation and at the control (i.e., IO_1_ vs. WCO_1_ and IO_2_ vs. WCO_2_); 2) medium and long-term effect, i.e., when the measurement is made by comparing the IO_1_ of the first evaluation with the IO_2_ and the WCO_2_ in the control session (i.e., IO_1_ vs. IO_2_ and IO_1_ vs. WCO_2_).

The comparisons were performed through *t*-tests between the mean values of the 11 considered quantitative parameters obtained per participant in the postural conditions listed above (IO_1_, IO_2_, WCO_1_, WCO_2_). Such a series of comparisons has been analyzed in terms of “*improvement,*” “*worsening,*” or “*unchanged*” concerning the original IO_1_ attitude measured at first evaluation.

Thus, each of the 11 postural parameters was classified as “*unchanged*” if no statistically significant difference between the two currently compared postural attitudes is found.

Conversely, we defined the following as “*improvement*”:

• *Frontal plane parameters:* When passing from the first to the second postural attitude (e.g., IO_1_ vs. WCO_1_, IO_2_ vs. WCO_2_, etc.), the parameter values approached the optimal theoretical zero value (D’Amico et al., 2018a).
• *Sagittal plane parameters:* In this case (except for pelvis torsion (|PT|) that should be zero), there are no theoretical optimal reference values, so we decided to consider the normative data determined in previous studies in healthy young adults as reference values to be approached (D’Amico et al., 2017b; D’Amico et al., 2018a; Kinel et al., 2018).
• *|∆UL| (i.e., the difference of underfoot load between the feet):* The optimal theoretical condition is achieved when there is a perfect balance of underfoot load distribution between the left and right sides; therefore, there was “*improvement*” when changes approached this condition passing from the first to the second postural attitude.

“*Worsening*”: Each time a statistically significant change differed from the definitions of “*improvement,*” it was concluded that a “*worsening*” had occurred.

A summarizing index was defined for each patient, assigning a +1, −1, or 0 scores when *improvement*, *worsening,* or *unchanged* was, respectively, determined (D’Amico et al., 2017b; D’Amico et al., 2018a; Kinel et al., 2021). Henceforth, a “global postural index” (GPI_i_) given by the sum of scores obtained for all the variables for the *i*th participant was defined. The frontal plane index (FPI_i_) and the sagittal plane index (SPI_i_) were defined by the sum of scores for the variables of the related group (Table 2).

Each of the summarizing indexes was regarded as “*improvement*” if the summed parameters got a positive score ≥50% of the maximum obtainable positive score; conversely, “*worsening*” if such sum got a negative score ≥50% of the maximum obtainable negative score; and “*unchanged*” in the other cases (D’Amico et al., 2018a; Kinel et al., 2021).

By counting the number of “*improvement,*” “*worsening,*” and “*unchanged*” obtained for each participant in each parameter, it is possible to determine the percentages of “*improvement,*” “*worsening,*” and “*unchanged*” achieved in each of the above-listed comparisons.

Finally, as in the group analysis, differences between NSLBP patients and healthy young adults [raw data from (D'Amico et al., 2017b)] were also investigated at the intrasubject level. Because there was no control session in the healthy young adults’ study, for such a group, only the IO_1H_ vs. WCO_1H_ could be included in the intrasubject analysis.

### 2.8 Power Analysis and Sample Size

The most critical condition among the various multivariate comparison tests is when NSLBP males vs. NSLBP females are compared in IO and WCO. Using GPower software (Faul et al., 2007), it can be established that being that the NSLBP patients’ sample composed of 32 males and 48 females, fixing the required power = 80%, α = 5%, and k = 11 (number of variates), the effect size is *d* = 1.00 (Mahalanobis distance).

Conversely, for Hotelling’s *T* ^2^ paired version, the IO_1_ vs. WCO_2_ test performed on 29 older NSLBP patients is the most critical condition. For such a case, *d* = 0.96 is the effect size.

## 3 Results

First of all, it is essential to underline the time-dependent action of LLD equalization on pain symptoms. Indeed, the NPRS average score was relatively high (NPRS = 7.8) at the first evaluation. In the medium-term follow-up group (4 months), the NPRS score dramatically decreased (NPRS = 1.1). The pain completely disappeared in the long-term (2 years) follow-up group (NPRS = 0).

The mean LLD optimal thickness found in the cohort of NSLBP patients slightly varied between the first evaluation and at control after the adaptation period. An LLD = 10.4 ± 6.2 mm for males and females LLD = 10.7 ± 7.2 mm was determined at the initial assessment. Conversely, at control, the LLD increased to 11.8 ± 6.6mm and 11.1 ± 8.4 mm for males and females, respectively. No statistical difference resulted between males and females.

### 3.1 Group Statistical Analysis

#### 3.1.1 NSLBP Patients’ Postural Characteristics and Reactions to LLD Equalization, Evaluated by Gender

In group statistical analysis, we investigated gender differences in the IO_1_ representing the initial indifferent orthostasis and the WCO_2_ representing the erect corrected standing posture outcome after the adaptation period to the LLD equalization provided by the customized foot orthotics (Table 3). The *T* ^2^ Hotelling test shows that there are differences between males and females in IO_1_, in particular the CA2 (second main CA), the SA (sacral angle), and the lumbar lordosis angle (LLA) present greater values in the females. At the control WCO_2_, only the LLA resulted greater in the females. Such a postural characteristic must be considered physiological because it is typically observed in the healthy young population in IO and WCO (D’Amico et al., 2017b).

**TABLE 3:** NSLBP Males vs. Females comparisons in IO_1_ and WCO_2_: Hotelling *T* ^2^ tests results, 95% confidence intervals and difference of means.

Hotelling *T* ^2^ test for independent samples NSLBP male: vs female in IO_1_and WCO_2_ comparison									
		IO_1_ (n1 = 32, n2 = 48, k = 11, T2 = 39.07, *p* = 3.7e-3, d = 1.42, power = 0.99)				WCO_2_ (n1 = 32, n2 = 48, k = 11, T2 = 37.0, *p* = 4.8e-3, d = 1.38, power = 0.98)			
Parameter	Descriptions	Males mean	Females mean	Difference in means	CI 95% lower÷upper	Males mean	Females mean	Difference in means	CI 95% lower÷upper
|ASO FR|(mm)	|Average frontal spinal Offsets|	8.0 ± 7.8	8.1 ± 5.6	0.03	−2.61÷2.68	4.5 ± 3.5	5.0 ± 4.3	0.50	−1.6÷2.6
|AGO FR|(mm)	|Average frontal global offsets|	11.6 ± 14.5	12.0 ± 8.7	0.34	−3.67÷4.34	5.3 ± 8.2	6.0 ± 7.2	0.76	−2.92÷4.43
CA1 (degrees)	1° Cobb angle	13.7 ± 9.7	15.6 ± 9.3	1.90	−2÷5.79	9.4 ± 7.5	10.4 ± 6.9	0.92	−2.64÷4.48
CA2 (degrees)	2° Cobb angles	8.4 ± 6.5	12.1 ± 8.9	**3.72** ^a^	0.14÷7.31	5.6 ± 4.2	7.7 ± 5.7	2.14	−0.61÷4.88
TKA (degrees)	“Thoracic” kyphosis angles	47.9 ± 18.1	47.1 ± 13.4	−0.74	−6.51÷5.02	47.5 ± 16.4	46.4 ± 12.6	−1.16	−7.65÷5.34
LLA (degrees)	“Lumbar” lordosis angles	35.3 ± 13.4	43.2 ± 9.8	**7.87** ^a^	3.46÷12.27	36.6 ± 15.5	42.3 ± 8.7	**5.72** ^a^	0.32÷11.12
|ΔASIS|(mm)	|∆Anterior superior iliac spine|	9.1 ± 7.5	10.3 ± 8.5	1.26	−2.22÷4.73	5.2 ± 5.1	6.2 ± 5.0	0.94	−1.69÷3.58
|ΔPSIS|(mm)	|∆Posterior superior iliac spine|	6.5 ± 3.4	6.7 ± 3.9	0.25	−1.44÷1.94	2.5 ± 2.9	2.6 ± 2.1	0.05	−1.39÷1.48
|PT|(mm)	|Pelvis torsion| = |(∆ASIS–∆PSIS)|	5.8 ± 4.9	6.1 ± 8.2	0.22	−2.95÷3.39	5.7 ± 6.0	6.6 ± 4.9	0.90	−1.89÷3.68
SA (degrees)	Sacral angle	14.5 ± 8.8	17.7 ± 8.2	**3.22** ^a^	0.3÷6.73	14.8 ± 8.3	17.3 ± 7.1	2.52	−1.31÷6.35
|ΔUL|(%BW)	|∆Underfoot load|	7.4 ± 5.0	8.3 ± 7.1	0.92	−2.66÷4.51	3.7 ± 2.8	4.7 ± 4.0	0.97	−1.1÷3.03

a associated with bold numbers indicates the statistically significant differences of means. IO_1_ (Indifferent Orthostasis at first evaluation); WCO_2_ (Wedge Corrected Orthostasis at control session).

The IO_1_ vs. WCO_1_ at first evaluation and IO_2_ vs. WCO_2_ at control sessions, after the adaptation period, evaluated by gender, provide information about the immediate-term postural reaction to LLD equalization (Table 4).

**TABLE 4:** Hotelling *T* ^2^ test for paired samples subdivided by gender and pooled data: IO_1_ vs. WCO_1_, IO_2_ vs. WCO_2_, IO_1_ vs. IO_2_, WCO_1_ vs. WCO_2_ and IO_1_ vs. WCO_2_.

Hotelling *T* ^2^ test for paired samples subdivided by gender and pooled data																
		Hotelling *T* ^2^ test for paired samples: per gender IO_1_ vs. WCO_1_ comparison			Hotelling *T* ^2^ test for paired samples: per gender IO_2_ vs. WCO_2_ comparison			Hotelling *T* ^2^ test for paired samples: per gender IO_1_ vs. IO_2_ comparison			Hotelling *T* ^2^ test for paired samples: per gender WCO_1_ vs. WCO_2_ comparison			Hotelling *T* ^2^ test for paired samples: per gender IO_1_ vs. WCO_2_ comparison		
Parameter	Descriptions	Males	Females	Pooled	Males	Females	Pooled	Males	Females	Pooled	Males	Females	Pooled	Males	Females	Pooled
|ASO FR|(mm)	|Average frontal spinal offsets|	—	—	—	**3.33**	**1.71**	**2.36**	—	—	—	—	—	—	**3.57**	**3.10**	**3.29**
|AGO-FR|(mm)	|Average-frontal-global-offsets|	—	—	—	**5.17**	**3.60**	**4.23**	—	—	—	—	—	—	**6.36**	**5.94**	**6.10**
CA1 (degrees)	1°-Cobb-angle	—	—	—	**1.76**	**1.76**	**1.76**	—	—	—	—	**2.91**	**2.28**	**4.26**	**5.24**	**4.85**
CA2 (degrees)	2°-Cobb-angles	—	—	—	—	—	**0.84**	—	—	—	**1.98**	**2.75**	**2.44**	**2.76**	**4.67**	**3.90**
TKA (degrees)	“Thoracic”-kyphosis-angles	—	—	—	—	—	—	—	—	—	—	—	—	—	—	—
LLA (degrees)	“Lumbar"-lordosis-angles	—	—	—	—	—	—	—	—	—	—	—	—	—	—	—
|ΔASIS|(mm)	|∆Anterior-Superior-iliac-spine|	—	—	—	**4.55**	**3.78**	**4.09**	—	—	—	—	—	—	**3.83**	**4.14**	**4.02**
|ΔPSIS|(mm)	|∆Posterior-superior-iliac-spine|	—	**4.02**	**3.98**	**2.86**	**3.86**	**3.46**	—	—	—	—	—	—	**3.93**	**4.13**	**4.05**
|PT|(mm)	|Pelvis-torsion| = |(∆ASIS–∆PSIS)|	—	—	—	—	—	—	—	—	—	—	—	—	—	—	—
SA (degrees)	Sacral-angle	—	—	—	**-0.76**	—	—	—	—	—	—	—	—	—	—	—
|ΔUL|(%BW)	|∆Underfoot-load|	**1.74**	**3.87**	**3.09**	**1.95**	—	**2.02**	**1.01**	—	—	—	—	—	**3.47**	**3.22**	**3.31**

All Hotelling *T* ^2^ paired samples tests resulted statistically significant. Signed differences of means: a positive difference indicates that the parameter mean value at the first tested condition is higher than the parameter mean value at the second tested condition; negative difference indicates the opposite (e.g. the 4.02 value for |ΔPSIS|(mm) in the IO_1_ vs. WCO_1_ test females column indicates that the |ΔPSIS| is higher in IO_1_ than WCO_1_; the −0.76 value for SA(degrees) in the IO_2_ vs. WCO_2_ males column indicates that SA(degrees) is higher in WCO_2_ than IO_2_.

Only statistically significant values of difference of means are showed in the table.

IO_1_ (Indifferent Orthostasis at first evaluation); WCO_1_ (Wedge Corrected Orthostasis at first evaluation); IO_2_ (Indifferent Orthostasis at control session); WCO_2_ (Wedge Corrected Orthostasis at control session).

The IO_1_ vs. WCO_1_ (immediate-term answer) shows that, at the beginning, at the group level, the statistically significant changes are few. Specifically, in the IO_1_ vs. WCO_1_, males present differences (improvements) only in |ΔUL| (underfoot load asymmetry). Conversely, in the females, the statistically significant changes (improvements) are in the |ΔUL| (underfoot load asymmetry) and |ΔPSIS| (pelvis obliquity as measured at the PSIS). When males and females are pooled together, the improvements are the same as in the females’ group.

More differences are evident in the immediate-term answer after the adaptation period. In the IO_2_ vs. WCO_2_ comparison, males show significant improvements in CA1, both the spinal and global frontal offsets (|ASO FR| and |AGO FR|), the pelvic obliquity (|ΔASIS| and |ΔPSIS|), the underfoot load asymmetry (|ΔUL|) and the SA. The same differences, except for SA, are found in females. When pooled together, in addition to the above parameters (excluding SA), also CA2 shows statically significant improvements.

The IO_1_ vs. WCO_2_ and the IO_1_ vs. IO_2_ and WCO_1_ vs. WCO_2_ (evaluated by gender) provide information about the structural postural changes induced by custom foot orthotics after the adaptation period. In the IO_1_ vs. WCO_2_ comparison, the results highlight clear statistically significant improvements in all the postural parameters of the frontal plane, including the underfoot load asymmetry (|ΔUL|), and it holds for all such parameters for males, females, and in the pooled females–males group as well (Table 4).

Conversely, the IO_1_ vs. IO_2_ comparison shows statistically significant improvement only in the underfoot load asymmetry (|ΔUL|) and only in men. In the WCO_1_ vs. WCO_2_ comparison, the improvements appear statistically significant in spine deformity angles (CA1 and CA2) for both genders.

The time-dependent (medium and long-term) and the age-dependent (older vs. young adults) effects of LLD equalization induced by customized foot orthotics are summarized in Table 5.

**TABLE 5:** Hotelling *T* ^2^ tests for paired samples for the IO_1_ vs. WCO_2_ comparisons of NSLBP patients subdivided by age and by short/long term.

Hotelling *T* ^2^ tests for paired samples IO_1_ vs. WCO_2_ comparisons of NSLBP patients subdivided by age and by short/long term					
Parameter	Descriptions	Young (*n* = 51)	Old (*n* = 29)	Short term (*n* = 40)	Long term (*n* = 40)
|ASO FR|(mm)	|Average frontal spinal offsets|	**2.33** ^a^	**3.70** ^a^	**2.45** ^a^	**4.12** ^a^
|AGO FR|(mm)	|Average frontal global offsets|	**4.70** ^a^	**5.88** ^a^	**6.86** ^a^	**5.35** ^a^
CA1 (degrees)	1° Cobb angle	**3.02** ^a^	**5.62** ^a^	**3.93** ^a^	**5.77** ^a^
CA2 (degrees)	2° Cobb angles	**1.73** ^a^	**5.54** ^a^	**3.04** ^a^	**4.77** ^a^
TKA (degrees)	“Thoracic” kyphosis angles	−0.38	−0.15	−0.77	1.89
LLA (degrees)	“Lumbar” lordosis angles	−0.19	0.27	−0.51	0.64
|ΔASIS|(mm)	|∆Anterior superior iliac spine|	**4.01** ^a^	4.21	2.56	**5.47** ^a^
|ΔPSIS|(mm)	|∆Posterior superior iliac spine|	**3.58** ^a^	**4.75** ^a^	**2.96** ^a^	**5.14** ^a^
|PT|(mm)	|Pelvis torsion| = |(∆ASIS–∆PSIS)|	−0.01	0.99	−0.97	0.45
SA (degrees)	Sacral angle	0.35	0.23	0.32	−0.02
|ΔUL|(%BW)	|∆Underfoot load|	**2.91** ^a^	4.88	2.48	**4.44** ^a^

All Hotelling *T* ^2^ paired samples tests resulted statistically significant. Signed differences of means: a positive difference indicates that the parameter mean value at the first tested condition is higher than the parameter mean value at the second tested condition; negative difference indicates the opposite (e.g. the 3.58 value for |ΔPSIS|(mm) in the IO_1_ vs. WCO_2_ test YOUNG, column indicates that the |ΔPSIS| is higher in IO_1_ than WCO_2_; the −0.01 value for |PT|(mm) in the same column indicates that |PT|(mm) is higher in WCO_2_ than IO_2_.

a associated with bold numbers indicates the statistically significant differences of means.

IO_1_ (Indifferent Orthostasis at first evaluation); WCO_2_ (Wedge Corrected Orthostasis at control session).

The medium-term results highlight clear statistically significant improvements in all the postural parameters of the frontal plane except for the underfoot load asymmetry |ΔUL| and the |ΔASIS| that require a more extended adaptation period as demonstrated by the long-term group in which also these two latter parameters become significantly improved.

The age-dependent (older vs. younger adults) comparison shows similar results to the time-dependent comparison. Indeed, the older adults group present statistically significant improvements in all the postural parameters of the frontal plane except for the underfoot load asymmetry |ΔUL| and the |ΔASIS|. Conversely, in the younger adults, all the postural parameters of the frontal plane present statistically significant improvements.

#### 3.1.2 Differences Between NSLBP Patients and Healthy Young Adults in Postural Characteristics and Reactions to LLD Equalization, Evaluated by Gender

The IO_1H_ vs. WCO_1H_ (immediate-term answer) shows that the female healthy young adults present significant improvements in both the frontal offsets |ASO FR| and |AGO FR|, in the pelvic obliquity by both the |ΔASIS| and |ΔPSIS|, and in the reduction of underfoot load asymmetry |ΔUL| (Table 6). The same improvements are also present for the healthy young males, but in this case, the |ΔASIS| and |ΔUL| do not reach statistical significance.

**TABLE 6:** NSLBP patients vs. healthy young adults subdivided by gender for the following comparisons: IO_1H_ vs. WCO_1H_, IO_1NSLBP_ vs. IO_1H_, WCO_1NSBLP_ vs. WCO_1H,_ and WCO_2NSBLP_ vs. WCO_1H_.

IO_1H_ vs. WCO_1H_: Hotelling *T* ^2^ test for paired samples (healthy males and females separately). IO_1NSLBP_ vs. IO_1H,_ WCO_1NSLBP_ vs. WCO_1H,_ WCO_2NSLBP_ vs. WCO_1H_: Hotelling *T* ^2^ test for independent samples (NSLBP patients vs. healthy young adults by gender comparisons)									
		IO_1H_ vs. WCO_1H_		IO_1NSLBP_ vs. IO_1H_		WCO_1NSLBP_ vs. WCO_1H_		WCO_2NSLBP_ vs. WCO_1H_	
Parameter	Descriptions	Males	Females	Males	Females	Males	Females	Males	Females
|ASO FR|(mm)	|Average frontal spinal offsets|	**2.48** ^a^	**3.03** ^a^	1.63	1.27	0.27	1.13	0.54	1.19
|AGO FR|(mm)	|Average frontal global offsets|	**8.88** ^a^	**10.04** ^a^	−0.35	−1.08	2.3	1.35	2.18	**3.03** ^a^
CA_1_ (degrees)	1° Cobb angle	1.02	−0.27	2.23	**5.22** ^a^	0.33	2.62	−1.01	−0.29
CA_2_ (degrees)	2° Cobb angles	0.67	0.43	0.23	**4.49** ^a^	−0.09	2.75	−1.77	0.11
TKA (degrees)	“Thoracic” kyphosis angles	−0.09	−0.28	2.27	−0.02	0.91	−1.23	1.88	−1.02
LLA (degrees)	“Lumbar” lordosis angles	−1.06	−0.01	2.11	−1.28	2.04	−1.04	2.27	−2.22
|ΔASIS|(mm)	|∆Anterior superior iliac spine|	0.78	**4.07** ^a^	1.51	1.45	0.14	1.31	−1.53	1.38
|ΔPSIS|(mm)	|∆Posterior superior iliac spine|	**3.78** ^a^	**3.46** ^a^	1.3	**1.90** ^a^	**1.18** ^a^	**1.35** ^a^	**1.16** ^a^	**1.23** ^a^
|PT|(mm)	|Pelvis torsion| = |(∆ASIS–∆PSIS)|	−1.49	0.99	0.35	0.42	0.8	**2.53** ^a^	−1.28	**1.94** ^a^
|ΔUL|(%BW)	|∆Underfoot load|	0.54	**1.87** ^a^	2.64	1.27	1.44	0.9	−0.51	0.97

All Hotelling *T* ^2^ paired samples tests resulted statistically significant. Signed differences of means: a positive difference indicates that the parameter mean value at the first tested condition is higher than the parameter mean value at the second tested condition; negative difference indicates the opposite (e.g. the 3.78 value for |ΔPSIS|(mm) in the IO_1H_ vs. WCO_1H_ test males column indicates that the |ΔPSIS| is higher in IO_1H_ than WCO_1H_; the −1.49 value for |PT|(mm) in the same column indicates that |PT|(mm) is higher in WCO_1H_ than IO_1H_.

a associated with bold numbers indicates the statistically significant differences of means.

IO_1_ (Indifferent Orthostasis at first evaluation); WCO_1_ (Wedge Corrected Orthostasis at first evaluation); IO_2_ (Indifferent Orthostasis at control session); WCO_2_ (Wedge Corrected Orthostasis at control session). Subscript H indicates Healthy young adults; Subscript NSLBP, indicates Nonspecific low back pain patients.

The IO_1NSLBP_ vs. IO_1H_ (evaluated by gender) provides information about the two groups’ structural postural characteristics. NSLBP females show significantly greater values in CA1, CA2, and |ΔPSIS| respect to healthy young women. Conversely, no differences are present for the males.

Comparing the immediate and medium-long term answers to LLD equalization, the NSLBP patients show residual difference only in |ΔPSIS| and |PT| for females and |ΔPSIS| for males. This happens in both WCO_1NSBLP_ vs. WCO_1H_ and WCO_2NSBLP_ vs. WCO_1H_ comparisons. Furthermore, it is possible to evaluate that the adaptation over time to LLD equalization reduces the differences between NSLBP patients and healthy subjects (Table 6).

#### 3.1.3 Correlation Between Heel Lift Thickness and Changes in CA1

At the first evaluation, the value of optimal thickness for LLD correction in NSLBP patients resulted in 10.4 ± 6.2 mm for males, 10.7 ± 7.2 mm for females, and 10.6 ± 4.8 mm pooled. No LLD statistical difference between genders has been found. At the control session, after the adaptation period, the LLD values presented a slight increase for males 11.8 ± 6.6 mm and females 11.1 ± 8.4 mm (still without statistical difference between genders). The mean main CA1 variation passing from IO_1_ to WCO_2_ is 4.8° ± 6.1°.

The correlation between the changes of the main CA1 passing from IO_1_ to WCO_2_ and the heel lift correction determined at first evaluation is low Tau = 0.17 but statistically significant (*p* = 0.032).

### 3.2 Intrasubject Statistical Analysis

**FIGURE 4:**
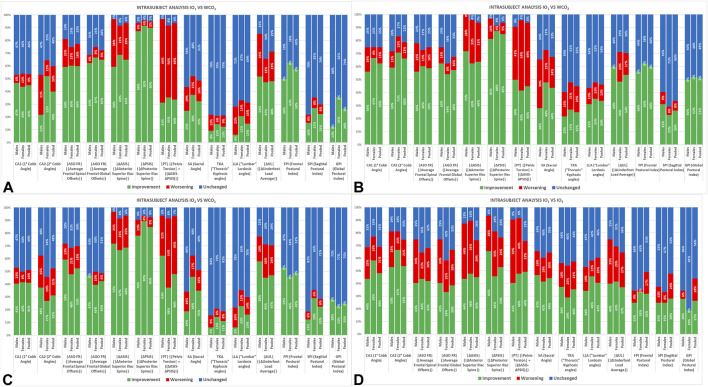
Intrasubject analysis subdivided by gender and pooled in NSLBP patient for the following comparisons: IO_1_ vs.WCO_1_ **(A)**, IO_1_ vs. WCO_2_ **(B)**, IO_2_ vs.WCO_2_ **(C)**, and IO_1_ vs. IO_2_ **(D)**. IO_1_ (IO at first evaluation); WCO_1_ (WCO at first evaluation); IO_2_ (IO at control session); WCO_2_ (WCO at control session).

**FIGURE 5:**
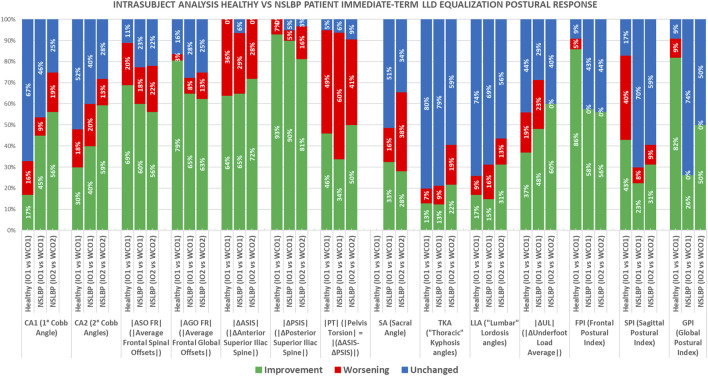
Intrasubject analysis healthy young adults’ vs. NSLBP patients’ pooled data immediate-term LLD equalization postural response comparisons: IO_1H_ vs. WCO_1H_, IO_1NSLBP_ vs. WCO_1 NSLBP_, IO_2 NSLBP_ vs. WCO_2 NSLBP_. Healthy young adults’ pooled data for the IO_1_ vs. WCO_1_ (data from (D’Amico et al., 2017b)). IO_1_ (IO at first evaluation); WCO_1_ (WCO at first evaluation); IO_2_ (IO at control session); WCO_2_ (WCO at control session). Subscript H indicates healthy young adults; subscript NSLBP indicates nonspecific low back pain patients.

Figures 4, 5 report the results of the intrasubject analysis.

The IO_1_ vs. WCO_1_ intrasubject analysis (Figure 4A) shows the immediate effect of the LLD equalization. Almost all frontal plane parameters and, additionally, |ΔUL| improve in both sexes (FPI shows “*improvements*” in 50% of males and 63% of females). Females show more “*improvements*” than males displaying higher percentages and favorable changes in a higher number of parameters.

Such results are consolidated at control after the adaptation period (IO_1_ vs. WCO_2_), in which males tend to reach the same effects as females (Figure 4B).

The IO_2_ vs. WCO_2_ intrasubject analysis (Figure 4C) is analogous to the IO_1_ vs. WCO_1_ but shows further “*improvements*” in pelvis torsion (|PT|).

In the IO_1_ vs. IO_2_ (Figure 4D), almost all parameters display higher percentages of “*improvements*” than “*worsening*” as witnessed by the summarizing indexes: FPI “*improvements*” (33% for females and 25% for males), SPI “*improvements*” (25% both for females and males), GPI “*improvements*” (19% for females and 28% for males).

Worth underlining with the LLD equalization, the “*worsening*” percentage for FPI and GPI equals zero in all the conditions IO_1_ vs. WCO_1_, IO_2_ vs. WCO_2_, IO_1_ vs. WCO_2_.

By analyzing the comparison with the healthy young adults (Figure 5), it results that, at immediate term, NSLBP patients improve more than the healthy subjects in those parameters in which they present the higher structural deviations from “healthy status,” such as CA1, CA2, and |ΔUL|, whereas the healthy individuals improve more on |ASO FR| and |AGO FR|.

## 4 Discussion

The present study aimed to define if LLD equalization treatment is effective in NSLBP patients to remove pain symptoms and structural-biomechanical asymmetries.

Eleven quantitative biomechanical parameters describing the nature of 3-D entire body posture were considered to study the gender, age-related, and time-dependent effects of LLD equalization treatment in NSLBP patients during 2 years of follow-up.

Our basic hypothesis was that the uncertainty about LLD measurement and treatment in LBP debated in the literature is mainly due to the lack of detailed, rigorous biomechanical-functional information on 3-D natural standing posture and gait characteristics and how the presence of an LLD influences them.

Indeed, given changes in multiple parameters that tend to occur with LLD (e.g., sacral or pelvic tilt and lumbar scoliosis, compensations in the lower limbs), it is likely that confounders are at play. Therefore, it is difficult to identify the true drivers of LBP in the LLD–LBP relationship (Sheha et al., 2018).

To tackle the several confounders at play, we used the nonionizing optoelectronic stereophotogrammetric approach associated with baropodometry presented in D’Amico et al. (2017a, 2017b). Such a method provides the evaluation of the 3-D entire skeleton posture, including the 3-D spine shape. In this way, it is easy and fast to quantify the eventual presence of an LLD and the whole skeleton functional postural adjustments when a heel-lift LLD correction is applied. As explained in the “Materials and Methods” section, to take into account both “anatomical” and “functional” LLD (Knutson, 2005a; Knutson, 2005b), the corrective wedge optimal size was determined as the degree of thickness that provided the best postural outcome for the individual in the frontal-plane parameters. Conversely, when it was impossible to achieve an overall improvement of the frontal-plane postural parameters, the optimal corrective-wedge thickness was chosen as the one providing the best equalization regarding the PSIS landmarks (D’Amico et al., 2017b).

In the present paper, the investigation has been limited to studying a 3-D entire skeleton standing posture in various conditions. No gait analysis has been performed.

At the first evaluation, the value of optimal thickness for LLD correction in NSLBP patients resulted in 10.4 ± 6.2 mm for males and 10.7 ± 7.2 mm for females. Such values are only about 1 mm higher than those found for healthy young adults (9.7 mm) in D’Amico et al. (2017b), and as in this previous study, no LLD statistical difference between genders has been found. Worth noting, at the control session, after the adaptation period, we found a slight increase of the LLD values resulting for males 11.8 ± 6.6 mm and females 11.1 ± 8.4 mm (still without statistical difference between genders). Such an outcome is likely to be connected to the pelvis torsion reduction after the LLD equalization adaptation period.

The patients were provided with customized foot orthotics incorporating a heel lift on the short leg side. The foot orthotics customization was supplied according to patients’ therapeutic needs, inducing LLD equalization and better biomechanical foot function (D’Amico et al., 2021b) (by considering the foot deformities and foot-floor interaction using baropodometry and 3-D foot scans). The heel lifts were corrective of the 100% LLD (D’Amico et al., 2012; D’Amico et al., 2017b) and were shaped from the calcaneus to the Lisfranc joint.

This study’s set of results leads to defining a coherent framework of postural responses to LLD equalization. A straightforward answer is that the heel-lift custom-made orthotics to equalize LLD is effective in NSLBP to remove pain symptoms and structural-biomechanical asymmetries.

The data confirm that the use of orthotics with 100% LLD equalization has no contraindications. In addition to the progressive total disappearance of painful symptoms, LLD equalization produces progressive improvements in postural parameters in the immediate, medium, and long terms. Specifically, the frontal plane parameters are the most strongly influenced by the orthotics corrections showing improvements for the majority of the subjects. Moreover, a not negligible percentage of NSLBP patients (31.3% of males and 22.9% of females) presented improvements in the sagittal plane after the medium-long term adaptation.

No patient presented any worsening in the frontal plane as witnessed by the FPI = 0% for “*worsening*” in each intrasubject comparison involving LLD equalization, i.e., IO_1_ vs. WCO_1_, IO_2_ vs. WCO_2_, IO_1_ vs. WCO_2_.

It is evident that there are immediate-term responses (IO_1_ vs. WCO_1_) to LLD equalization stimulus reducing unbalancing and asymmetries; medium-term (IO_1_ vs. WCO_2_ 4 months follow up group), and long-term (IO_1_ vs. WCO_2_ 2 years follow up group) responses that lead to structural changes.

From the group statistical analysis (confirmed at intrasubject level), on the gender, age-related, time-dependent effects of LLD equalization treatment, it can be noted that the adaptation times are individual, being connected to the relative joint mobility of the pelvis (in particular the sacroiliac joint) and spine. For such reasons, there could be some light differences between genders.

For example, the differences between genders in NSLBP patients are evaluated in two occurrences (Table 3): i.e., through the comparison of the indifferent orthostasis at the first evaluation (IO_1_) and through the comparison of the wedge-corrected orthostasis at the follow up (WCO_2_). In both assessments, the females tended to have more significant asymmetries and spinal deformities than males even if such difference does not reach the statistical significance except for CA2 in IO_1_ (Table 3).

The value of LLA is significantly higher in women than in men as it happens for healthy young adults (D’Amico et al., 2017b) in both IO_1_ and WCO_2_.

In any case, at follow-up (WCO_2_), both the genders converge toward a similar final improved and more balanced standing erect posture. Starting from a worse postural condition, the women show a more marked improvement, particularly spinal deformities reduction.

The intrasubject statistical analysis (Figure 4) confirmed the difference between the sexes in IO_1_ vs. WCO_1_ and IO_1_ vs. WCO_2_. Indeed, the women present a higher percentage of “*improvements*” in almost all frontal plane values (or positive changes in a higher number of parameters) as witnessed by the summarizing indexes starting from the immediate effect (FPI shows “*improvements*” in 50% of males and 63% of females in IO_1_ vs. WCO_1_). Even in the sagittal plane, females perform better than males. Such results are consolidated at control after the adaptation period when the IO_1_ vs. WCO_2_ shows “*improvements*” in most patients, and males tend to reach the same results as females. Some parameters show a faster answer to the LLD equalization as it happens to |ΔPSIS| (WCO_1_), and some others need a more extended adaptation period leading to a higher percentage of “*improvements*” at control (WCO_2_) as it happens for |ΔASIS| and so |PT|, CA1, CA2. The pelvis obliquity at posterior-superior iliac spine (|ΔPSIS|) level disappeared for more than 85% of subjects with the women improving in a more significant percentage than men (women 88%, men 81% of improvements). Conversely, only about 66% of the patients achieved the anterior-superior iliac spine (|ΔASIS|) leveling. In this case, men present a more significant percentage of improvements than women (women 63%, men 72%). Women demonstrated a better reduction of the spinal deformities (67% vs. 56% for primary CA1 and 71% vs. 59% for secondary CA2). For the underfoot load asymmetry (|ΔUL|), an overall improvement is obtained for 54% of subjects (women 49%, men 60%). This condition is also confirmed by the global body side-leaning (|AGO FR|), which globally improved in 58% of the sample (women 54%, men 63%). Regarding the trunk side-leaning (|ASO FR|), the results showed improvements in 59% of subjects (women 60%, men 56%).

Regarding the time-dependent LLD equalization effects, it is possible to see that the immediate response (IO_1_ vs. WCO_1_) induces high percentages of improvements in the intrasubject analysis (Figure 4) in almost all the frontal plane parameters (especially in women). However, such high percentages are not enough to obtain a general statistical significance in the group analysis. Indeed, only |∆UL| for both genders and |∆PSIS| for females are significantly different (Table 4), thus indicating the considerable variability of individual responses.

Conversely, in the medium–long term LLD equalization (i.e., IO_1_ vs. WCO_2_), the effects after the adaptation period show the convergence to an overall statistically significant improvement for all the frontal parameters at group level in males and females. The most relevant improvements as highlighted by Hotelling’s *T* ^2^ (Table 4) are the reductions of spinal deformities (CA1: 5.24° females 4.26° males, 4.85° pooled, and CA2: 4.67° females, 2.76° males, 3.9° pooled), underfoot load asymmetry (|∆UL|: -body weight percentage - 3.22% females, 3.47% males, 3.31% pooled) and pelvis horizontality realignment (|∆PSIS|: 4.13 mm females, 3.93 mm males, 4.05 mm pooled, and |∆ASIS|: 4.14 mm females, 3.83 mm males, 4.02 mm pooled).

Such behavior is likely related to structural changes in the pelvis, spine, proprioception, and motor control that induce better postural balance and more symmetrical distribution of underfoot loads.

A further understanding of how the medium–long term equalization acts structurally in modifying the standing posture can be obtained by associating the results of the following group comparisons (Table 4) IO_1_ vs. WCO_2_, IO_1_ vs. IO_2_, and WCO_1_ vs. WCO_2_.

In the IO_1_ vs. IO_2_ comparison, only the underfoot load asymmetry (|ΔUL|) is statistically significant and only for men. Whereas in the WCO_1_ vs. WCO_2_ comparison, improvements are statistically significant only in spine deformity angles (both the CA1 and CA2) for both genders.

Such outcomes, considered together with the IO_1_ vs. WCO_2_ comparison, demonstrate that the improvements obtained after the adaptation period using the customized foot orthotics determine significant changes in the postural structure of the body and also that these structural changes are maintained only through the continuous use of LLD and foot posture corrections. Otherwise, the patients’ posture tends to return to the initial conditions.

Such structural changes modify the immediate term answer to LLD equalization as it is possible to evaluate by considering the IO_1_ vs. WCO_1_ and the IO_2_ vs. WCO_2_ comparisons. Indeed, the IO_1_ vs. WCO_1_ comparison displays that the immediate response to LLD equalization acts significantly only on the |∆UL| and |∆PSIS| parameters. Conversely, in IO_2_ vs. WCO_2_ (which represents the immediate response to LLD equalization at control after the adaptation period) the same parameters, also significant in the IO_1_ vs. WCO_2_ comparison, are statistically significant.

This set of outcomes leads us to conclude that the adaptation period induces progressive changes in joint mobility and neuromotor control, particularly at the pelvis and spine level. This is achieved through the various functional activities (walking, standing in various conditions, etc.) performed while wearing the customized corrective insoles, inducing changes in body balance and proprioception, stimulating new motor control strategies. These changes modify the response to the stimulus in those parts of the body that showed structural alterations at the first evaluation (spinal deformities, torsion of the pelvis, etc.). The |∆UL| is the only parameter in which the motor control system shows a memory of the “adaptation-learning” phenomenon even without LLD equalization as highlighted by the IO_1_ vs. IO_2_ comparison. When the stimulus is applied again (i.e., in the IO_2_ vs. WCO_2_), the response involves the entire structure, inducing significant changes in all the frontal plane postural parameters and partially also in sagittal plane parameters. Therefore, it is evident that equalization acts on the whole-body structure and the neuromotor system. It is also evident that when the stimulus is missing, the posture returns to the initial state of imbalance (IO_1_ vs. IO_2_). Thus, LLD equalization needs some adaptation time (the duration of which varies on an individual basis) to be effective on the entire body posture, and it must be maintained over time to prevent the body structure from returning to its original asymmetric unbalanced state. Indeed, if LLD equalization is removed, the process of progressive formation of structural deformities and asymmetries restarts.

Other aspects of the coherent framework of postural responses to LLD equalization derive from the analysis of young vs. older adults’ behavior and the medium- vs. long-term adaptation period.

From Table 5, it is possible to highlight that older adults start from conditions of more significant postural asymmetries and spinal deformities. LLD equalization induces significant improvements in all frontal plane parameters in younger adults, including the underfoot load asymmetry (|∆UL|). Conversely, LLD equalization induces improvements of greater magnitude in older adults than in younger adults though they reach the statistical significance in a reduced number of parameters.

Comparing the medium to the long-term adjustment period shows that the longer, the better as the long-term group reaches the statistical significance in all the frontal plane parameters, including the |∆UL| (Table 5). Thus, confirming the need for an adaptation period long enough to obtain structural posture changes.

Comparing healthy young adults and NSLBP patients adds another piece to the jigsaw puzzle.

Interestingly, the comparison of indifferent orthostases (i.e., the IO_1H_ vs. IO_1NSLBP_) between healthy young adults and NSLBP patients shows very few differences in the natural upright posture. NSLBP men present no significant difference compared with healthy young adults. In contrast, women display significant differences in spinal deformities and |ΔPSIS| parameters. Minimal differences between healthy young adults’ and NSLBP patients’ postures are also confirmed when LLD equalization is applied. In such a condition, the differences between healthy young subjects and NSLBP patients tend to vanish in almost all the considered parameters except for residual difference only in |ΔPSIS| and |PT| for females and |ΔPSIS| for males (Table 6). Worth noting such convergence is already at the immediate term response, i.e., WCO_1H_ vs. WCO_1NSLBP_ and successively confirmed at medium–long term WCO_1H_ vs. WCO_2NSLBP_ when the adaptation over time to LLD equalization produces the effect of a further reduction of the residual differences between NSLBP patients and healthy subjects.

Comparing the intrasubject performance of healthy young adults and NSLBP patients shows that, in the short term, the latter present higher percentages of “*improvements*” compared with healthy subjects in those parameters in which the structural deviations from the “healthy status” are the most remarkable (Figure 5), such as CA1, CA2, and |ΔUL|. Healthy young adults show higher rates of “*improvements*” on |ASO FR| and |AGO FR| and a very high percentage of “*unchanged*” in the sagittal plane. In such latter plane, patients with NSLBP show better performance with relevant percentages of “*improvements.*”

LLD is reported as relatively common in the literature, affecting up to 90% of the population with an average value of 5.2 ± 4.1 mm as measured by highly precise radiographic millimetric methods (Knutson, 2005a). The relationship between LLD and LBP is an open debate. LLD is correlated with asymmetrical distribution and magnitude of mechanical stresses and strains in spine joints, degenerative changes in the lumbar spine, alterations in spinal biomechanics, and LBP (Giles, 1981; Friberg, 1982; Friberg, 1983; Gofton, 1985; Helliwell, 1985; Rossvoll et al., 1992; ten Brinke et al., 1999; Defrin et al., 2005; Golightly et al., 2007; Kendall et al., 2014; Murray and Azari, 2015; Cambron et al., 2017; Menez et al., 2021; Menez et al., 2020).

LLD is associated with some degree of pelvic tilt in the coronal plane and very often with pelvis torsion in the sagittal plane, deriving from distinct inter-ASIS and inter-PSIS height differences. Pelvis torsion is defined as an intrasegmental pelvic pattern in which one ilium is more tilted toward the anterior than the other (Beaudoin et al., 1999; Knutson, 2005a; D’Amico et al., 2017b).

Authors Gurney (2002) and Raczkowski et al. (2010) describe that alterations in pelvis symmetry, the tilt on the frontal plane, and the 3-D pelvis torsion induce changes in the sacrum attitude, producing variations in the dynamics of the lumbar vertebrae and possibly developing LBP and lumbar scoliosis with a higher probability to present convexity toward the short limb (Giles and Taylor, 1982; Murray et al., 2017; Sheha et al., 2018; Gordon and Davis, 2019). Even mild extents of LLD can change pelvic posture (Betsch et al., 2012, 2013; Kwon et al., 2015; D’Amico et al., 2017b). This latter can be associated with several postural compensations. These compensations may include foot pronation and/or hip and knee flexion on the longer limb, foot supination and/or hip and knee extension on the shorter limb. These induce muscle imbalance, which may cause several dysfunctions at various levels such as the sacroiliac joint (Perttunen et al., 2004), hip flexor contracture on the long limb, or short limb plantar flexor contracture (Raczkowski et al., 2010).

Such compensations induce biomechanical-functional changes in the foot–ground interaction that must be corrected with customized insoles.

As a consequence, the coexistence, in most cases, of anatomical and functional contributions adds difficulties to the LLD evaluation. Among the clinical methods, the tape method has been criticized. Indeed, potential sources of error of such a technique relate to differences in leg circumference, angular deformities, and difficulty in accurately palpating bony prominences as well as joint contractures (Cleveland et al., 1988; Beattie et al., 1990; Rondon et al., 1992; Terry et al., 2005; Sabharwal and Kumar, 2008). In contrast, the use of standing blocks under the short leg to level the pelvis is more reliable and complete than tape measurement by giving the possibility also to consider the LLD functional component but not as accurate as imaging modalities. However, even on imaging tools to be used, there is a debate, and they present pros and cons, especially thinking about the risk connected to X-ray use (Sabharwal and Kumar, 2008) for some of them.

The magnitude of LLD likely plays a role in LBP although it is unclear what degree of LLD is required to cause symptoms (Friberg, 1982; Friberg, 1983; Defrin et al., 2005; Golightly et al., 2007; Kendall et al., 2014; Murray and Azari, 2015; Cambron et al., 2017; Menez et al., 2021; Menez et al., 2020). Some investigators have tried to quantify a significant LLD, accepting as much as 20 (Gross, 1978) to 30 mm (Reid and Smith, 1984) as the minimal difference to be corrected, whereas others define a significant discrepancy in terms of functional outcomes (Abraham and Dimon, 1992). Various authors find postural unbalancing and disorders associated with LLD lower than 10 mm (Giles and Taylor, 1976; Giles and Taylor, 1982; Friberg, 1983; Defrin et al., 2005; D’Amico et al., 2012).

However, there is still no convergence of opinions leading to the shared definition of treatment guidelines (Sheha et al., 2018). For such a reason, more recently, it has been suggested that “it must be discussed with each patient individually whether the treatment should be conservative or surgical. The extent of the discrepancy is not the sole determining factor for the mode of treatment. The decision to treat is always elective” (Vogt et al., 2020).

Indeed, also body functional activity likely plays a role in the LLD–LBP relationship. Rannisto et al. (2015) established that workers with LLD who have to stand for many hours for their work activity are more likely to have LBP than seated workers. This result looks pretty reasonable, especially from the biomechanical point of view, as the LLD can induce high load asymmetries and, therefore, high asymmetrical stresses that can be distributed at the spine level leading to postural imbalances and spinal deformities as our results describe.

It is worth noting that the same group (Rannisto et al., 2019) conducted a randomized controlled study on those standing work activity workers (meat cutters) presenting LBP associated with LLD. They obtained reduced subjective pain and the probability of taking sick leave days by applying an LLD heel lift of 5 mm or more.

Even the best corrective thickness of shoe lifts is a matter of debate. Indeed, there is no agreement on the LLD measurement method and the rationale to apply for the correction given the difficulty of evaluating its global influence on standing posture from a functional point of view. Studies present the following approaches: lifts equal to the amount of LLD (Raczkowski et al., 2010; D’Amico et al., 2012; D’Amico et al., 2017b), lifts few millimeters less than the amount of LLD (Friberg, 1982), lifts equal to LLD minus 10% (Chuter et al., 2014; Ashour et al., 2019), a lift of 50% of LLD (Menez et al., 2021; Menez et al., 2020), and lifts that caused resolution of LBP symptoms (Golightly et al., 2007). It is not a surprise that the strategies for lift application were variable. Researchers provide lifts to correct LLD completely at once (Gurney, 2002; D’Amico et al., 2012; D’Amico et al., 2017b), whereas others (Defrin et al., 2005; Golightly et al., 2007) provided lifts that were gradually adjusted over time.

The quantitative functional evaluation of posture helps to overcome all the above issues. Indeed, it highlights a strong relationship between LLD and postural unbalancing associated with NSLBP and allowed to find the optimal LLD equalization.

Given the individual biomechanical-functional compensations due to LLD and the individual responses to LLD equalization as also witnessed by the low correlation between heel lift thickness and changes in main CA1, identifying the minimum LLD to treat is an “ill-posed” problem. Indeed, the same LLD can produce different outcomes for subjects with different anthropometric, proprioception, and motor control characteristics. Thus, the treatment must be individualized through appropriate measurement approaches.

The proposed approach helps to overcome also the controversial debate on functional vs. anatomical LLD nature (Knutson, 2002; Knutson, 2005a; Knutson, 2005b). The optimal LLD correction induces overall postural parameter improvement, disregarding either the functional or anatomical origin of LLD.

The suitable heel rise correction was included in the customized orthotics to fix the foot functional behavior using the complementary information derived from baropodometry and 3-D foot scan. Furthermore, the present study demonstrates that the application of the correction equal to 100% of the measured LLD, which was included in the orthotics soon after the first evaluation in a complete way (i.e., without starting with smaller thicknesses, which were progressively increased up to the final desired thickness), led to improvements without any overall worsening or adverse effects. Quick pain relief responses to LLD equalization have been noted in the agreement with many other studies (including also randomized control studies) (Friberg, 1982; Friberg, 1983; ten Brinke et al., 1999; Defrin et al., 2005; Golightly et al., 2007; Kendall et al., 2014; Murray and Azari, 2015; Cambron et al., 2017; Sheha et al., 2018; Rannisto et al., 2019; Menez et al., 2021; Menez et al., 2020) up to the complete pain remission after an adaptation period, the duration of which is strictly individual. Personalized therapy/rehabilitation treatments would surely help to speed up and stabilize the recovery process (Giles and Taylor, 1976). Such treatment should consider the clinical and postural evaluation and focus on increasing joint mobility and muscle strength to reduce spinal deformities and improve proprioception and motor control to equalize the asymmetric muscular efforts associated with LLD.

Studying the posture in healthy young adults, D’Amico et al. (2017b) established that “to be asymptomatic does not mean to have an optimal posture. It seems that asymmetry (associated with LLD unbalanced postural and underfoot loads, spinal curvature in the frontal plane, and pelvis torsion) is standard in both sexes.” The comparison of the NSLBP sample with the healthy young adult population showed that the postural-structural characteristics of the two groups were very similar except for the presence of more pronounced spinal deformities in NSLBP females and generally of a slightly higher LLD (1–2 mm) in NSLBP patients. Depending on individual morphological-physiological characteristics and proprioceptive and motor control traits, the prolonged condition of imbalance due to LLD could lead to several postural-functional compensations, accelerating degenerative processes leading to musculoskeletal injury disorders and spinal deformities accentuation (Friberg, 1983; Defrin et al., 2005; Tallroth et al., 2005; Harvey et al., 2010; Kendall et al., 2014; Murray and Azari, 2015; Tallroth et al., 2017; Sheha et al., 2018; Gordon and Davis, 2019). We also observed that older NSLBP patients presented greater asymmetry values than the younger NSLBP patients. The long-term LLD equalization produced better postural outcomes than the immediate and medium-term. Such results bring us to strengthen the hypothesis, presented many times in literature that there is likely a biomechanical component in the development of NSLBP and other spinal and lower limb musculoskeletal disorders, such as hip or knee osteoarthritis (Chuter et al., 2014; Cambron et al., 2017; Campbell et al., 2018; Sheha et al., 2018; Gordon and Davis, 2019; Rothschild et al., 2020). However, as demonstrated, if the LLD is equalized, the degenerative process can be slowed down or even reversed, at least from a postural-functional point of view.

The set of results of the present study provide strong support to the hypothesis that even asymptomatic healthy young people with LLD, asymmetrical underfoot load distributions, and associated mechanical stresses and strains in spine joints, over time, could evolve into alterations in spinal biomechanics, inducing degenerative changes in the lumbar spine and, therefore, develop LBP or other pathologies. Such considerations confirm the hypothesis of D’Amico et al. (2017b) that “as a rule of thumb, it could be argued that to prevent eventual possible long term negative consequences of asymmetry due to musculoskeletal imbalance on joints and spine, clinicians could apply a suitable form of LLD equalization and prescribe regular/periodic performance of focused physical activity, in order to reduce asymmetry in the subject.”

A limitation of this study is that the analysis of the postural response to LLD equalization in the medium and long term was conducted by pooling together people of different ages not separated by gender although we verified the existence of different responses due to both sex and age. Similarly, the analysis of the postural response to LLD equalization in younger and older adults was conducted by grouping together medium and long-term follow-up NSLBP patients not separated by gender. Such a choice was due to the limited size of the sample. Such kind of further detail will be a matter of future study. In the literature, it is suggested that gait and range of movements analysis for patients with LBP provide better functional-clinical observation and treatment (Bratton, 1999). In previous papers, we describe how the proposed methodology, also in association with surface electromyography, can quantitatively detail the spine range of movement and the affected flexion-relaxation phenomenon in the trunk forward bending as well as a thorough evaluation of gait characteristics in LBP patients (D’Amico et al., 1995b; D’Amico et al., 2008; D’Amico et al., 2021a). Such evaluations were not included in the present study. This will be a matter for future research.

## 5 Conclusion

Heel-lift customized orthotics with 100% LLD correction are an effective short- and long-term treatment in patients with NSLBP, inducing complete recession of pain symptoms and stimulating postural parameter improvement without contraindications. LLD equalization needs some adaptation time, the duration of which varies on an individual basis, to be effective on the entire body posture. Such benefits are maintained if the customized orthotics are donned. The interruption of their use can lead to progressive regression with the return to the starting postural conditions.

The 3-D stereophotogrammetry is demonstrated to be a helpful tool to perform the postural measurement, quantifying LLD magnitude and the LLD effects on whole skeleton posture and spine deformities, correction, and monitoring. Indeed, it highlights a strong relationship between LLD and postural unbalancing associated with NSLBP and allows finding the optimal LLD equalization.

Given the individual biomechanical-functional compensations due to LLD and the individual responses to LLD equalization, identifying the minimum LLD to treat is an ill-posed problem. The same LLD can produce different outcomes for subjects with different anthropometric, proprioception, and motor control characteristics. Thus, the treatment must be individualized through appropriate measurement approaches.

Healthy young adults and NSLBP patients show very few differences in the natural upright posture. Minimal differences between healthy young adults’ and NSLBP patients’ postures are also confirmed when LLD equalization is applied, showing the tendency to vanish in almost all the considered parameters.

## Data Availability

The raw data supporting the conclusion of this article will be made available by the authors, without undue reservation.

## Footnotes

1 Bioengineering and Biomedicine Company Srl Pescara-Italy.

2 NaturalPoint Inc. Corvallis, OR-USA.

3 Zebris Gmbh Isny-Germany.

## Abbreviations

IO, indifferent orthostasis; LBP, low back pain; LLD, leg length discrepancy; NSLBP, nonspecific low back pain; WCO = Wedge corrected orthostasis.
